# Percutaneous coronary intervention vs coronary artery bypass grafting in the management of chronic stable angina: A critical appraisal

**DOI:** 10.1016/s0975-3583(10)12003-8

**Published:** 2010

**Authors:** Alok Kumar Singh

**Affiliations:** *Department of Cardiology, C S M Medical University, Lucknow - 226 003, India*

**Keywords:** Coronary artery bypass grafting, percutaneous coronary intervention, chronic stable angina, revascularization

## Abstract

Chronic stable angina is a clinical expression of myocardial ischemia associated with fixed atherosclerotic coronary stenosis, which prevents the adaptation of coronary circulation resulting in an increased oxygen requirement. We recommend that once the diagnosis of chronic stable angina is made, first every patient should be offered the optimal medical therapy, including ACE inhibitors, beta-blockers, statins, and nitrates. If the patients' symptoms are not controlled in spite of these drugs being used in maximum tolerated dosages, then these patients should be subjected to coronary angiography. If a patient shows a single-or double-vessel disease, then PCI should be offered. On the contrary, if the coronary angiogram shows a triple-vessel disease and left main disease, then one has to look for comorbidities that put the patient at a higher risk of CABG and the patient should be treated with PCI. Other patients with left main and triple-vessel disease having diabetes and left ventricular dysfunction should go directly for surgical revascularization. Overall, health related quality of life (HRQoL) is similar in both PCI and CABG.

Coronary artery disease (CAD) remains a major global public health problem. Chronic stable angina is a clinical expression of myocardial ischemia associated with fixed atherosclerotic coronary stenosis, which prevents the adaptation of coronary circulation to an increased oxygen requirement. It is more common in men than in women and its prevalence increases sharply with age. About one half of patients presenting at the hospital with myocardial infarction (MI) have preceding angina.[1] On this basis, it has been estimated that there are 30 patients with a stable angina for every patient with infarction who is hospitalized. The annual[2,3] rate of MI is 3–3.5% and the annual mortality is 2–3%.

The goals of treatment include relief of symptoms, inhibition or slowing of disease progression, prevention of future cardiac events, such as MI, and improved survival. Here we focus on the role of revascularization strategies [percutaneous coronary intervention (PCI) vs coronary artery bypass grafting (CABG)] in the management of chronic stable angina.

Major changes in the revascularization strategies of symptomatic obstructive CAD have been seen in the past decade with a substantial shift toward PCI. It is always a matter of heated discussion as to which revascularization strategy is better. Although both the strategies have their merits, they are complementary to each other and should not replace one another. In Table 1, the relative advantages of both the strategies have been summarized.

**Table 1:** Advantages and disadvantages of PCI vs CABG

	PCI	CABG
Age	Preferred in very old and very young age	Avoided in extremes of age
Comorbidities	PCI preferred in	CABG is preferred in
	-Acute coronary syndrome	Diabetes
	-Severe pulmonary diseases	Left ventricular systolic dysfunction
	-Dementia	History of bleeding
	-High stroke risk	Aspirin/clopidogrel allergy
Anatomical consideration	PCI treats focal lesion	CABG replaces whole artery and hence offers
	Preferred in low syntax scores	complete revascularization
		Preferred in high syntax scores
Patients preference/clinical factors	Less invasive	More invasive
	No scar mark	Scar mark
	Shorter hospitalization	Longer hospitalization
	Early work resumption	Late work resumption
	Easily repeatable	Redo CABG carries high mortality/morbidity
	More chances of recurrence of angina	Less chances of recurrence of angina

PCI, percutaneous coronary intervention; CABG, coronary artery bypass grafting

## AMERICAN COLLEGE OF CARDIOLOGY/AMERICAN HEART ASSOCIATION REVASCULARIZATION GUIDELINES

We have summarized the recommendations from the American College of Cardiology/American Heart Association (ACC/AHA, 2002) practice guidelines for chronic stable angina,[4] which were unchanged in the 2007 update[5] and generally similar to those from the unstable angina/non–ST-elevation MI guidelines[6] from 2007 and have been summarized in Table 2.

**Table 2:** ACC/AHA 2007 revascularization guidelines[5]

	PCI	CABG
1- or 2-Vessel disease with mild or no symptoms, mild or no ischemia on noninvasive testing, and not yet receiving adequate medical therapy	III	III
1- or 2-Vessel disease with moderate area at risk and ischemia on noninvasive testing	IIA	IIA
1- or 2-Vessel disease with a large area at risk, noninvasive test indicating high risk, or failure of medical therapy	I	I
3-Vessel disease or 2-vessel disease + pLAD, without diabetes mellitus or congestive heart failure	I	I
3-Vessel disease or 2-vessel Disease + pLAD, and diabetes mellitus or congestive heart failure	IIB	I
LM stenosis > 50% in a candidate for CABG	III	I
LM stenosis > 50% in a noncandidate for CABG	IIB	N/A

LM,; pLAD; PCI, percutaneous coronary intervention; CABG, coronary artery bypass grafting

## MEDICAL THERAPY VS PERCUTANEOUS CORONARY INTERVENTION

PCIs are being increasingly used in patients with various manifestations of CAD. They represent an established treatment strategy that improves survival and survival free of recurrent MI in patients with ST-segment elevation MI. Early invasive therapy also improves long-term survival and reduces late MI in patients with non–ST-segment elevation MI. Although PCI reduces symptoms in patients without acute coronary syndromes, its effects on the prognosis of these patients are still not defined. The assessment of this issue has been difficult for at least 2 reasons. First, patients with a stable CAD have a very good prognosis and large sample size studies are required to assess the potential differences in treatments in the case of rare events. All studies performed till date were far from having sufficient power to assess mortality, and also, there is a certain risk associated with PCI, which leads to the aggregation of events in a relatively short period after the procedure. Any potential beneficial effect of PCI compared with medical treatment alone may require time to offset this early aggregation of adverse events.

In Courage Trial, 2287 patients who had an objective evidence of myocardial ischemia and significant CAD were randomized to PCI with optimal medical therapy.[7] The 4.6-year cumulative primary-event rates were 19.0% in the PCI group and 18.5% in the medical therapy group (hazard ratio for the PCI group, 1.05; 95% confidence interval [CI], 0.87–1.27; *P* = 0.62). There were 211 primary events in the PCI group and 202 events in the medical therapy group. There were no significant differences between the PCI group and the medical therapy group in the composite of death, MI, and stroke (20.0% vs 19.5%; hazard ratio, 1.05; 95% CI, 0.87–1.27; *P* = 0.62); hospitalization for acute coronary syndrome (12.4% vs 11.8%; hazard ratio, 1.07; 95% CI, 0.84–1.37; *P* = 0.56); or MI (13.2% vs 12.3%; hazard ratio, 1.13; 95% CI, 0.89–1.43; *P* = 0.33). The authors of Courage Trial have concluded that, as an initial management strategy in patients with stable CAD, PCI did not reduce the risk of death, MI, or other major cardiovascular events when added to optimal medical therapy.

Recently, a meta-analysis of 17 trials, including the Courage Trial by Schömig *et al*.[8] have shown that in chronic stable angina patients, the PCI strategy in comparison with that of the medical treatment group, leads to a 20% reduction in the odds ratio (OR) of all-cause death (OR: 0.80; 95% CI: 0.64–0.99, *P* = 0.263 for heterogeneity across the trials). Allocation to the PCI group was associated with a nonsignificant 26% reduction in the OR of cardiac death (OR: 0.74, 95% CI: 0.51–1.06). In the PCI group, 319 patients had a nonfatal MI after randomization compared with 357 patients in the medical treatment group (OR: 0.90, 95% CI: 0.66–1.23). Findings from this meta-analysis suggest that a PCI-based invasive strategy may improve long-term survival compared with a medical treatment-only strategy in patients with stable CAD.

## PERCUTANEOUS CORONARY INTERVENTION VS CORONARY ARTERY BYPASS GRAFTING

A number of large randomized trials in the 1990s directly compared CABG with PCI. Their major finding was that survival was similar for the 2 modes of management, although PCI was associated with more repeated interventions.

One important exception was that patients with insulin-requiring diabetes had a significantly higher 5-year survival rate after CABG than after PCI (BARI Trial).[9]

Meta-analysis[10] of trials conducted before 1995, when coronary stenting was rare, revealed no significant differences in the treatment strategies for either death or the combined endpoint of death and MI. Mortality during the initial hospitalization for the procedure occurred in 1.3% of the CABG group and 1% of the PCI group. The need for subsequent revascularization was significantly higher in the PCI group, and although patients were significantly less likely to have angina 1 year after the bypass surgery than after PCI, by 3 years this difference was no longer statistically significant. Results from the BARI study, the largest single randomized trial of PCI vs surgery, not included in this meta-analysis, were nonetheless consistent with these findings, although a survival advantage with bypass surgery was observed in the diabetics.

It should be noted that similar to comparisons of PCI and pharmacotherapy, the early trials did not use stents or internal mammary artery grafts. These limitations were overcome in the ARTS I and SoS randomized trials comparing CABG with mostly arterial grafts to PCI with stent implantations. The ARTS I Trial[11] compared the strategy of multiple-stent implantation with the aim of complete revascularization vs bypass surgery in patients with multivessel disease. However, this trial was not carried out exclusively among patients with stable angina; 37% and 35%, respectively, in both arms, had unstable angina, 57% and 60%, respectively, had stable angina, and 6% and 5%, respectively, had silent ischemia. As in the previous analyses of balloon angioplasty, at 1 year, there was no difference between the 2 groups in terms of rate of death, stroke, or MI. Among patients who survived without stroke or MI, 16.8% of those in the stenting group underwent a second revascularization, when compared with 3.5% of those in the surgery group. The rate of event-free survival at 1 year was 73.8% among the patients who received stents and 87.8% among those who underwent bypass surgery. As measured 1 year after the procedure, coronary stenting for multivessel disease in selected patients offered a similar outcome in terms of death, stroke, and MI as bypass surgery. However, stenting was associated with a greater need for repeated revascularization.

The ARTS II registry[12] indicated that the solution to revascularization may lie in the use of drug-eluting stents. The rate of major adverse cardiac and cerebrovascular events in this study was similar to that of the CABG arm in the ARTS I Trial and significantly lower than that of the PCI with bare metal stent arm. After adjusting for risk factors, the authors noted a lower rate of major cardiovascular and cerebrovascular events in the PCI arm of ARTS II than in the CABG arm of ARTS I. However, the treatment assignment was not random. Most of the patients included in these randomized trials were relatively at low risk—fewer than 20% had left ventricular dysfunction and almost 70% had 1- or 2-vessel disease—and therefore, were compatible with the patient group from whom CABG had not been found to be superior to PCI therapy. CABG has an advantage over PCI, which does not detect unstable plaques or the lesions most likely to be the cause of subsequent cardiac events.

A meta-analysis by Hoffman *et al*., including trials of stents,[13] suggests a mortality benefit with CABG compared with PCI at 5 years, which continued up to 8 years in patients with multivessel disease, as well as significantly less angina and less need for a repeat revascularization. However, observational data with a 3-year follow-up on 60,000 patients from the New York Cardiac Registry[14] indicated that for patients with 2 or more diseased coronary arteries, CABG was associated with higher adjusted rates of long-term survival than stenting.

A meta-analysis by Bravata *et al*.[15] in 2007, which included 23 RCTs in which 5019 patients were randomly assigned to PCI (POBA and BMS) and 4944 patients were randomly assigned to CABG showed that CABG was superior to PCI in relieving angina and averting repeat revascularization procedures. The rates of survival at 1, 5, and 10 years were similar for both the procedures, even though CABG carried a higher risk of stroke (1.2% vs 0.6% with PCI). However, most of the 23 studies did not involve patients with severe CAD (ie, left main or 3-vessel CAD). A limitation of this meta-analysis is that the author did not assess how the comparative outcomes vary according to clinical factors, such as the extent of coronary disease, ejection fraction, or previous procedures, and a majority of the patients randomized in the meta-analysis were not subjected to the latest revascularization technique.

Furthermore, PCI carries a risk for subacute and late-stent thrombosis and a need for prolonged antiaggregation treatment. A recent trial comparing PCI with CABG in patients with multivessel disease (ERACI–III) reported a higher rate of stent thrombosis (3.1% in 18 months). These data indicate that both the revascularization techniques are appropriate and comparable in outcome, except in patients with left main, 3-vessel disease, and in diabetics for whom CABG is currently preferred.[16]

Similar to this, in a more recent meta-analysis by Daeman *et al*.[17] of 4 randomized trials (ARTS, ERACIII, MASS-II, and SoS Trials), PCI with stenting was associated with a long-term safety profile similar to that of CABG in multivessel CAD. However, as a result of persistently lower repeat revascularization rates in the CABG patients, the overall major adverse cardiac and cerebrovascular event rates were significantly lower in the CABG group at 5 years.

Recently, the result of the Synergy between PCI with Taxus and Cardiac Surgery (SYNTAX) Trial,[18] in which 1800 patients with left main or 3-vessel CAD were randomly assigned to undergo CABG or PCI (with drug-eluting stents) to determine which was the better revascularization strategy.

In the SYNTAX Trial, patients treated with PCI involving drug-eluting stents were more likely than those undergoing CABG to reach the primary endpoint of the study—death from any cause, stroke, MI, or repeat revascularization—within 12 months after randomization (17.8% of patients vs 12.4%). In an analysis of secondary endpoints, the 2 treatment groups had similar rates of death from any cause, stroke, or MI (7.6% for PCI and 7.7% for CABG). Patients undergoing PCI were more likely than those undergoing CABG to require repeat revascularization (13.5% vs 5.9%) but were less likely to have a stroke (0.6% vs 2.2%). This trial did not directly compare both the strategies in all the chronic stable angina patients as it was only anatomy guided. But even in a high-risk anatomy group, patients with serious coexisting conditions or vessels unsuitable for grafting (about 5% of patients in the SYNTAX study) are poor candidates for CABG, and they should be encouraged to undergo PCI. If safe and complete revascularization is possible with either PCI or CABG as it was in approximately 60% of the patients in the SYNTAX study, an assessment of coronary anatomy should be performed and a SYNTAX score assigned.[19] The presence of complex coronary anatomical features (indicated by a high SYNTAX score) identifies patients with an increased risk of a suboptimal outcome with PCI; and they should be encouraged to undergo CABG.[20] Conversely, patients with less complex coronary anatomy (ie, a low SYNTAX score) should be presented with the advantages and disadvantages of each procedure and allowed to choose between them.[21]

Recently, in a nonrandomized study, including 240 patients in CABG group and 229 patients in PCI group, the health-related quality of life (HRQoL) and the change in the NYHA class after CABG or PCI in the management of stable CAD have been assessed.[22] A three-year survival in both the groups was similar; HRQoL improved statistically in both the groups until 6 months after treatment but deteriorated toward the end of the follow-up of 36 months. Clinically evident improvement of the HRQoL and decrease of the NYHA class took place more frequently among the CABG patients. Despite the initially more serious preoperative morbidity, the CABG patients achieved an equal level of HRQoL when compared with the PCI patients. The CABG patients might also obtain better relief from symptoms in the mid-term follow-up.

Coronary artery disease remains a major global public health problem. Chronic stable angina is a clinical expression of myocardial ischemia associated with fixed atherosclerotic coronary stenosis, which prevents the adaptation of coronary circulation resulting in an increased oxygen requirement. As per the data available today, we recommend that once the diagnosis of chronic stable angina is made, every patient should be offered the optimal medical therapy, including ACE inhibitors, beta-blockers, statins, and nitrates. If the patients' symptoms are not controlled in spite of these, then these patients should be subjected to coronary angiography. If a patient shows a single-or double-vessel disease, then PCI should be offered. On the contrary, if the coronary angiogram shows a triple-vessel disease and left main disease, then one has to look for comorbidities that put the patient at a higher risk of CABG and the patient should be treated with PCI. Other patients with left main and triple-vessel disease having diabetes and left ventricular dysfunction should go directly for surgical revascularization. Otherwise, in the patients of left main and triple-vessel disease, SYNTAX score should be calculated; if it is high (60%), then they should directly proceed for surgical revascularization, if low (1/3 patients), then either of the strategy can be offered as the initial method of revascularization. Overall, HRQoL is similar in both PCI and CABG.
